# Alcohol Use in China: Unrecorded and Recorded Bai Jiu in Three Rural Regions

**DOI:** 10.3390/ijerph19010405

**Published:** 2021-12-30

**Authors:** Lanyan Ding, Baoping Song, Chengli Wu, Ian M. Newman, Lok-Wa Yuen, Ling Qian, Botao Wang, Wenjuan Zhang, Ping Wei

**Affiliations:** 1 Center for Mental Health Education, Xidian University, Xi’an 710126, China; lyding@xidian.edu.cn (L.D.); botaowang@xidian.edu.cn (B.W.); wjuanzhang@xidian.edu.cn (W.Z.); pwei@xidian.edu.cn (P.W.); 2 Nebraska Prevention Center for Alcohol and Drug Abuse, University of Nebraska-Lincoln, Lincoln, NE 68588, USA; inewman1@unl.edu (I.M.N.); lok-wa.yuen@huskers.unl.edu (L.-W.Y.); 3 Department of Guidance and Training, Chinese Center for Health Education, Beijing 100011, China; zxzx@mail.xidian.edu.cn

**Keywords:** grain spirits, distilled spirits, bai jiu, noncommercial alcohol, unrecorded alcohol, drinking patterns, alcohol preferences

## Abstract

In China, approximately 70% of beverage alcohol is consumed in the form of spirits. An estimated 25% of all alcohol consumed is unrecorded, mostly spirits (bai jiu), produced outside regulatory systems in small neighborhood distilleries, mostly in rural areas. Unrecorded bai jiu drinkers are generally older, male, prefer higher-strength bai jiu, and drink daily and mostly at home. To explore possible regional differences, researchers used interview data from 2919 bai jiu drinkers in rural areas in Hebei, Anhui, and Hubei provinces in China. Results confirmed that patterns varied by province. The sample in Hubei preferred unrecorded bai jiu with a more stable preference to alcohol type, tended to drink less frequently, and reported experiencing less drinking pressure, suggesting lower-risk drinking patterns in this region. The Hebei and Anhui sample reported higher frequency and greater amount of alcohol consumption, were more likely to experience drinking pressure, indicating higher-risk patterns in alcohol use in these two regions. The results provide needed details about regional differences in unrecorded bai jiu drinking patterns that are not evident in aggregated data and suggest variations in drinking patterns that may reflect local geography, local values, traditions, and ethnic differences.

## 1. Introduction

Beverage alcohols of all types play an important but unspectacular role in Chinese life. Alcohol serves as a key component of diet and medicine and is an iconic symbol for hospitality and special celebrations. In China, it is widely believed that alcohol, in small amounts, is beneficial for health. Few laws regulate the sale or use of alcohol, and enforcement seems inconsistent. The one exception is the law relating to drunk driving, which is taken seriously and enforced regularly [1,2].

The World Health Organization (WHO) estimated for 2018 that 68.6% of males and 42.6% of females in China consumed alcohol in the past 12 months. Among the drinkers, the estimated alcohol per capita consumption was 17.0 L of pure alcohol for males and 6.0 for females. Chinese drinkers overwhelmingly prefer to drink distilled spirits (67.2% of per capita recorded alcohol consumption) over beer (29.6%), wine (3.1%), or other alcoholic beverages (<1%) [3] (p. 356). In addition, China has a uniquely intricate etiquette and drinking culture around distilled spirits.

In China, a significant portion of the distilled spirits (called bai jiu in Chinese) that are consumed are unrecorded. The unrecorded bai jiu discussed in this paper is distilled from grains and produced in rural homes and small local distilleries. Qian and Newman [4] described unrecorded bai jiu production and noted that most unrecorded bai jiu is sold and consumed within the same local social network in which it is made. This type of alcohol, made using traditional methods, is sometimes called artisanal alcohol. The amount of unrecorded bai jiu produced annually by individual families and small distilleries is relatively small, but a large number of these artisanal distilleries in China means the combined contribution to total per capita alcohol consumption is a significant but unknown amount.

Compared to recorded bai jiu that is produced in licensed factories and is taxed, unrecorded bai jiu is usually produced, sold, and consumed outside of official alcohol sale or tax records and is not subject to routine testing or inspections. With no regulation or quality test, unrecorded bai jiu is considered by some public health authorities to be a possible threat to health [5,6]. In addition, because unrecorded bai jiu is not taxed and not regulated, the alcohol industry argues that it has an unfair economic advantage in the marketplace [7,8]. Increasing our understanding of unrecorded bai jiu use would help alleviate public health authorities’ concerns on its safety and the alcohol industry’s concerns on its unfair (untaxed) advantage in the market.

A recent systematic review of unrecorded alcohol consumption around the world noted a “dearth of information on China” [9] (p. 884). Slowly this information void is being filled. There is now a detailed description of the production of unrecorded alcohol in rural areas [4]. Analyses of samples of unrecorded bai jiu [10,11,12] suggest it is no more dangerous than recorded alcohol.

This conclusion is similar to a finding from studies of unrecorded distilled spirits collected in Europe [5,6,9]. Two meta-analyses of alcohol use by young people in China suggested geographic differences in alcohol use [13,14]. Wei et al. [15] described important differences between users of recorded and unrecorded distilled spirits in rural areas, noting that unrecorded bai jiu drinkers were older, male, preferred higher alcohol-by-volume (ABV) bai jiu, were more likely to drink daily, and more likely to drink at home. They chose unrecorded bai jiu because it was cheap, they preferred the taste and appreciated the local, traditional (artisanal) product, but they said they were willing to switch to recorded bai jiu if given the opportunity.

In a country as large and diversified as China, differences among regions and provinces could not be ignored when planning for policy and public health programs. This analysis of data from rural areas in three provinces contributes to the developing body of knowledge by reporting differences between two groups of drinkers (unrecorded and recorded bai jiu drinkers) at the province level. This analysis contributes new information by assisting in the reduction in the void in information about unrecorded bai jiu use in China.

One research question guided this study:

What are the alcohol use patterns among unrecorded and recorded bai jiu drinkers in three rural regions in China?

## 2. Materials and Methods

### 2.1. The Research Sites

**Figure 1:**
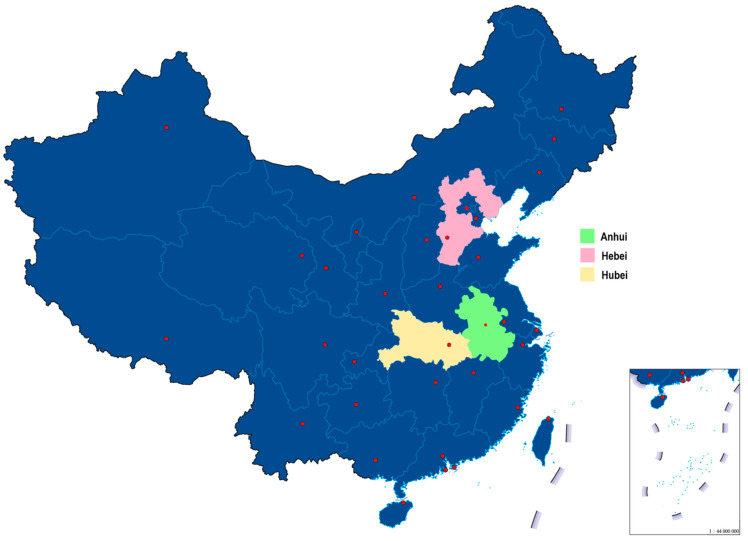
A map indicating the location of Anhui, Hebei, and Hubei in China. This map is provided by Shanghai Baotu Internet Techonology Company with an authorization [16].

Data about unrecorded and recorded bai jiu consumption were collected in the rural areas of three provinces: Anhui, Hebei, and Hubei, which are indicated in a map in Figure 1 [16]. The provinces were selected based on reports that unrecorded alcohol was easily available and based on previously established contacts with local authorities that minimized administrative obstacles.

#### 2.1.1. Anhui

Anhui is located in the North China Plain and borders Jiangsu on the north and east, Zhejiang on the southeast, Jiangxi to the south, and Hubei and Henan to the west. The central part of the province is fertile and densely populated. Wheat and sweet potatoes are grown in northern Anhui, and rice and wheat in southern Anhui. Once considered a poor agricultural province, the presence of coal, iron and copper has led to considerable wealth concentrated in the regions close to the Yangtze River. The rural residents interviewed for this study came from the southwest, northwest, northeast, and north-central parts of Anhui. In 2020, 65.75% of Anhui’s population was aged 15–64, and 15.01% was 65 and older. Almost half (41.67%) of the population were rural residents, and 4.49% of Anhui’s population age 15 or above were illiterate, 26.88% were at primary or below educational level, 33.72% were junior high, 13.29% were senior high, 13.28% were at college or above level. In 2020, the total production of recorded bai jiu is 282,113.3 kiloliters [17].

#### 2.1.2. Hebei

Hebei is north of the Yellow River and borders the dual metropolises of Beijing and Tianjin. Hebei’s agricultural products include cereal grains, cotton, peanuts, soybeans, and sesame. The rural residents interviewed for this study came from the central and southern parts of Hebei. In 2020, 65.85% of Hebei’s population was aged 15–64, and 13.92% was 65 and older. About 39.93% of the population were rural residents, and 1.51% of Hebei’s population aged 15 or above were illiterate, 24.66% were at primary or below educational level, 39.95% were junior high, 13.86 were senior high, 12.41% were at college or above level [17].

#### 2.1.3. Hubei

Hubei borders Shaanxi and Henan in the north, Anhui to the northeast, Jiangxi to the southeast, Hunan, and the municipality of Chongqing to the west (Figure 1). Blessed with abundant water, Hubei is sometimes called the “land of fish and rice”. Wheat, tea, and cotton are important products, as well as a range of minerals from the province’s mines. The rural residents interviewed for this study came from the eastern and central parts of Hubei. In 2020, 69.10% of Hubei’s population was aged 15–64, and 14.59% was aged 65 and older. About 37.11% of the population were rural residents, and 2.32% of Hubei’s population aged 15 or above were illiterate, 23.52 were at primary or below educational level, 34.28% were junior high, 17.43% were senior high, 15.50% were at college or above level [17].

### 2.2. Sampling

The present study aimed to investigate the pattern of bai jiu drinking in rural China, especially unrecorded bai jiu drinking. Interview sites were selected based on reports of widespread use of unrecorded bai jiu in rural areas. In total, 21 villages were involved: eight in Anhui, eight in Hebei, and five in Hubei. Only residents over the age of 18 who had consumed alcohol in the last year were eligible for this study.

### 2.3. Interview Strategy

Data were gathered by face-to-face interviews to accommodate the range of literacy among the rural residents and to provide an opportunity for data-gatherers to explain the definition of alcohol use. Besides its being a regular part of socializing, alcohol has other daily uses in some parts of China, so alcohol survey participants may not be aware of their consumption in casual use, medicinal use, or meal-related drinking. Interviewers could ask follow-up questions to prompt participants to list all alcohol use [15].

Before beginning, interviewers explained the purpose of the interview, and participants were told they could decline to answer any questions and could leave the interview at any time without any negative consequences. Alcohol use is not illegal for any age group. All those interviewed signed a consent form. Data were gathered by trained graduate public health/epidemiology students. Each interview took approximately 15 min.

### 2.4. Questionnaire Development

A questionnaire for investigating noncommercial alcohol drinking behavior among rural residents was developed based on previous research in China [18,19,20,21,22,23,24,25]. The survey consisted of 39 items, including 6 questions for demographic information and 33 questions for drinking behaviors, including drinking frequency, drinking quantity, the type of alcohol, most memorable drinking experience, preference for recorded or unrecorded bai jiu, the experience of pressuring others to drink or themselves being pressured to drink. Examples of items for investigating drinking behaviors are “did you drink in the last year” and “why did you choose unrecorded bai jiu”. Survey questions were developed and pretested under the supervision of a working group including Chinese local authorities, public health researchers in China, and epidemiologists in the United States.

### 2.5. Defining Unrecorded and Recorded Bai Jiu Drinkers

For this study, membership in the unrecorded or the recorded bai jiu drinking group was based on the type of alcohol a person consumed on daily drinking occasions. Types of alcohol included in the interview question were unrecorded bai jiu, recorded bai jiu, beer, yellow wine, rice wine, grape wine, and fruit wine. Unrecorded bai jiu drinkers were those who usually drank unrecorded bai jiu exclusively or with beer and wine. Recorded bai jiu drinkers were those who usually drank recorded bai jiu exclusively or with beer and wine. Those who did not consume any bai jiu were excluded from this analysis.

The participants who reported they drank both unrecorded and recorded bai jiu on a “usual drinking” occasion were sorted by their answer to a subsequent question about the proportion of the total amount of bai jiu they drank that was unrecorded bai jiu (1/4 or less, 1/2, 3/4, or all). If the majority (3/4) of their total bai jiu consumption was unrecorded bai jiu, the participant was classified as an unrecorded bai jiu drinker. If “1/4 or less” was unrecorded bai jiu, the participant was classified as a recorded bai jiu drinker. Because our objective in this analysis was a clear view of differences between patterns of use of recorded and unrecorded bai jiu, participants who reported no clear preference (1/2) were eliminated from the analysis.

### 2.6. Quantity and Frequency

Estimating alcohol quantity and frequency of consumption in China is difficult. Alcohol is sold in containers of a variety of sizes, served in many different types of (nonstandard) cups, comes in a wide range of strength (ABV), and unrecorded bai jiu, in particular, may not be labeled. On social drinking occasions, people at the table frequently “top up” one another’s drinks, pour drinks of varying sizes, and space drinks close together or farther apart as the social occasion unfolds. Competitive drinking situations can reduce a person’s recall of quantities and types of alcohol consumed. We followed the strategy developed by Wei and colleagues for classifying quantity and frequency responses [15]. For quantity, interviewees were asked to estimate the number and size of bottles of beer, and to estimate the quantity of wine and bai jiu (recorded and unrecorded) in liang, a commonly used measure (one liang is approximately 50 g). Wei [15] noted that unrecorded bai jiu drinkers tended to drink the same type of alcohol with the same ABV, usually at home, at regular times during the day. They suggest this increases the accuracy of unrecorded bai jiu drinker recall. Their field observations suggested older men are the most accurate in estimating the quantity and reporting frequency of unrecorded bai jiu use. For frequency, interviewers asked drinkers to estimate how many days in a typical week they drink, ranging from every day, 3–6 days a week, and <3 days a week.

### 2.7. ABV on Memorable Occasions

In China, people are more likely to drink in the home of a friend or relative or in the company of friends or relatives [25]. They usually drink to establish, strengthen, and maintain relationships on memorable occasions, which involve alcohol use at festivals, ceremonies, and banquet events. Drinking at these celebratory occasions aiming at socialization may bring a different meaning than drinking alone. On memorable occasions, bai jiu drinkers were divided into two groups according to the answer to the question for the strength of alcohol they consumed: the group who drank high-strength bai jiu (≥42% ABV) on memorable occasions and the group who drank low-strength bai jiu (<42% ABV) on memorable occasions.

### 2.8. Stability of Preference for Alcohol Type

Stability of preference was determined by the identification of unrecorded vs. recorded bai jiu drinkers and their preference of alcohol type when given a free choice. For example, if unrecorded bai jiu drinkers said they would choose unrecorded bai jiu when they could have any drink they wanted, they were considered to have a stable preference. If unrecorded bai jiu drinkers said they would choose recorded bai jiu or an alcoholic beverage other than bai jiu when they could choose freely, they were classified as having an unstable preference.

### 2.9. Statistical Analysis

The demographic characteristics of unrecorded and recorded bai jiu drinkers in the three provinces were described, and their associations were indicated by chi-square tests. Considering that participants in the same village are more likely to share variability due to the similarity in traditional cultures and geographical vicinity, multilevel modeling was necessary to account for the dependency of responses. Level 1 (individual level) estimated the influence of unrecorded bai jiu drinking when controlling for the differences in sex, age, education, and occupation. Level 2 (villages level) assessed the influence of regional differences. Subject to the nature of outcomes, different types of models were performed. Two-level logistic regressions with random intercept were applied to test the effect of province on unrecorded bai jiu drinking, strength of alcohol consumed on memorable occasions, stability of drinking preference, and being pushed and pushing others to drink. A two-level multinomial logistic regression was used to test the effect of province on drinking frequency. Two-level linear regressions were used to test the effect of province on drinking quantity on daily and memorable occasions. All analyses were conducted with Stata 15 for Windows.

### 2.10. Ethical Considerations

The Institutional Review Board for the Use of Human Subjects at the University of Nebraska—Lincoln approved the project (approval #: 20120412640 EX). Additional approvals were obtained from Tongji Medical College and the relevant authorities in each province.

## 3. Results

### 3.1. Sample Characteristics

A total of 3298 drinkers were recruited in the selected interview sites, and 3268 interviews were successfully completed (99.1%): Hubei = 1110, Anhui = 1070, Hebei = 1088. We excluded 24 respondents who did not consume any bai jiu in the last year as well as 325 respondents who had no clear preference between recorded and unrecorded bai jiu from the analysis, leaving a sample of 2919 bai jiu drinkers: 924 in Anhui (21.8% females, 78.1% males), 946 in Hebei (14.4% females, 85.6% males), and 1049 in Hubei (10.6% females, 89.4% males). All the participants were over the age of 18. Of the sample, 1592 (54.5%) were classified as recorded bai jiu drinkers, and 1327 (45.5%) were classified as unrecorded bai jiu drinkers: a majority of them were in Hubei (66.4%), 25.4% were in Anhui, and 8.2% were in Hebei (χ^2^ (2) = 1097.48, *p* < 0.001). Within each province, there was no sex difference in either unrecorded or recorded bai jiu drinkers, but unrecorded and recorded bai jiu drinking was associated with age, education, and occupation (Table 1).

### 3.2. Province and Unrecorded Bai Jiu Use

A two-level logistic regression was used to test the effect of province on unrecorded bai jiu consumption, controlling for differences in sex, age, education, and occupation. Compared to bai jiu drinkers in Hubei Province, the odds of being an unrecorded bai jiu drinker were lower for Anhui (OR = 0.06, *p* < 0.001) and Hebei (OR = 0.01, *p* < 0.001). Hubei Province was selected for the reference group here (and below) because of its reputation for high unrecorded bai jiu production and consumption (Table 2).

### 3.3. Province and Drinking Frequency

We conducted a two-level multinomial logistic regression to test the effect of province on drinking frequency. Unrecorded bai jiu drinkers had higher odds of drinking every day versus less than three times a week (OR = 3.52, *p* < 0.001) and higher odds of drinking three to six times a week versus less than three times a week (OR = 2.31, *p* < 0.001) than recorded bai jiu drinkers. The Hebei sample showed similar odds of drinking every day versus less than three times a week (OR = 1.34, *p* = 0.231) but lower odds of drinking three to six times a week versus less than three times a week (OR = 0.32, *p* < 0.001) than the Hubei sample, holding the demographic variables and unrecorded bai jiu drinking constant. The odds of drinking every day (OR = 1.03, *p* = 0.917) and the odds of drinking 3–6 times a week (OR = 0.68, *p* = 0.097) were not significantly different between drinkers in Anhui and Hubei (Table 3).

### 3.4. Province and Drinking Quantity

Two-level linear regressions were used to test the effect of province on the quantity of bai jiu consumed on daily drinking occasions as well as on memorable occasions (celebratory drinking), controlling for differences in the demographic variables. The quantity of bai jiu consumed did not differ between unrecorded and recorded bai jiu drinkers at both daily drinking (Table 4) and memorable occasions (Table 5). However, on memorable occasions, both Anhui and Hebei samples consumed more bai jiu than the Hubei sample (Anhui: *β* = 0.94, S.E. = 0.43, *p* = 0.040; Hebei: *β* = 1.99, S.E. = 0.44, *p* < 0.001).

### 3.5. Province and Strength of Alcohol Consumed on Memorable Occasions

Participants were divided into the group who drank high-strength bai jiu (≥42% ABV) on memorable occasions and the group who drank low-strength bai jiu (<42% ABV) on memorable occasions. Results of a two-level logistic regression indicated a significant difference between unrecorded and recorded bai jiu drinkers in the strength of the bai jiu they drank on memorable drinking occasions (OR = 1.60, *p* = 0.001). Compared to the Hubei sample, the odds of consuming high-strength vs. low-strength bai jiu were lower for the Anhui (OR = 0.18, *p* = 0.015) and Hebei (OR = 0.05, *p* < 0.001) samples when the demographic variables and unrecorded bai jiu drinking were held constant (Table 6).

### 3.6. Province and Stability of Preference for Alcohol Type

Stability of drinkers’ preference was invested in seeing if drinkers would prefer the same alcohol type when given a free choice. Results of a two-level logistic regression indicated that the unrecorded bai jiu drinkers had lower odds of preferring the same alcohol type than recorded bai jiu drinkers (OR = 0.25, *p* < 0.001). Compared to the Hubei sample, the odds of preferring the same alcohol type were lower for the Anhui (OR = 0.54, *p* = 0.020) and Hebei (OR = 0.46, *p* = 0.004) samples when the demographic variables and unrecorded bai jiu drinking were held constant (Table 7).

### 3.7. Province and Drinking Pressure

In China, being pushed by other people to drink and pushing others to drink are common social interactions related to hospitality, friendship, and friendly competition.

Results of two-level logistic regressions indicated that the unrecorded bai jiu drinkers had a higher likelihood of being pushed to drink than recorded bai jiu drinkers (OR = 1.64, *p* < 0.001) (Table 8), while the odds of pushing others to drink was also higher for unrecorded bai jiu drinkers (OR = 1.56, *p* < 0.001) (Table 9). More importantly, compared to the Hubei sample, the odds of being pushed to drink and pushing others to drink were higher for the Anhui sample (being pushed: OR = 2.88, *p* < 0.001; pushing: OR = 2.17, *p* = 0.004) and Hebei sample (being pushed: OR = 2.24, *p* = 0.007; pushing: OR = 1.75, *p* = 0.040) (Table 8 and Table 9).

## 4. Discussion

In China, rural households often made bai jiu from their own harvest, or they could buy or trade it from a neighborhood distiller. Practically all villages have at least one family whose main source of income is distilling bai jiu, making bai jiu readily available for consumption. The supply of bai jiu, because it is a locally produced product, is yet subject to many factors, such as local geography, farming, and wealth. Local cultural practices and traditions may affect the patterns of use, as could bai jiu’s role in the daily diet and its role as medicine, its importance in festivals and special ceremonies, and its role in hospitality.

This paper compares the patterns of use of unrecorded and recorded bai jiu in three rural regions in China. Results confirmed that patterns varied by province (1) residents in rural Hubei preferred unrecorded bai jiu with higher-strength, reported a more stable preference of alcohol type, and experienced less drinking pressure than their counterpart in the other two regions, indicating regional drinking patterns under the possible cultural controls, (2) the Hebei and Anhui sample reported heavier drinking and were more likely to experience social pressure in drinking, suggesting higher-risk drinking patterns that were possibly caused by local customs and drinking etiquette.

The odds of being an unrecorded bai jiu drinker were significantly higher for residents in Hubei than Anhui and Hebei. This finding is consistent with other large alcohol surveys [26,27]. Hubei includes a lake-studded alluvial plain along the middle reaches of the Yangtze River with reliable rain and a well-developed irrigation system. The availability of water in Hubei supports extensive rice growing, which guarantees rice is always available and cheap, likely increasing unrecorded bai jiu production and, subsequently, consumption.

Unrecorded bai jiu distillers often pride themselves on the strength of their bai jiu. Similarly, individual drinkers pride themselves on their ability to drink high-ABV bai jiu. In this sample, unrecorded bai jiu drinkers more likely consumed high-ABV bai jiu than recorded bai jiu drinkers, and in Hubei, more drinkers drank high-ABV bai jiu on memorable occasions than those in the other two provinces. Unrecorded bai jiu was most common in Hubei, and knowing that Hubei drinkers are more likely to select high-ABV beverages raises a question for regional risk. This might be especially risky for the percentage of people in China who inherited a genetic trait for impaired acetaldehyde metabolism [10,11].

Other research suggested bai jiu drinkers chose unrecorded bai jiu because it was cheaper, because of its tradition, and because it tastes better [4,15]. We assessed the importance of these and other qualities by asking respondents if given free choice, what type of bai jiu would they select on their next drinking occasion. Unrecorded bai jiu drinkers were more likely to switch to recorded bai jiu compared to the recorded bai jiu drinkers. In Hubei Province, unrecorded bai jiu drinkers were less likely to switch than the unrecorded bai jiu drinkers in Anhui and Hebei province. This suggests Hubei Province, whose drinkers preferred unrecorded bai jiu with a more stable preference of alcohol type may value unrecorded bai jiu differently.

Being pushed and pushing others to drink is common and considered an indication of friendship. Unrecorded bai jiu drinkers are more likely to be pushed and to push others to drink than recorded bai jiu drinkers, suggesting this behavior is associated with the traditional unrecorded bai jiu rather than the more recently introduced recorded alcohol. Interestingly, the odds of pushing and being pushed were lowest in Hubei Province, where there was a preference for high-ABV drinking and a lower likelihood of switching to recorded bai jiu. In most parts of China, Confucianism continues to be the basis of many practices and beliefs in daily life. Central to this is respect for others and for an explicit social order. Confucian values also proposed that for alcohol, there was no limit, but that there should be no bad behaviors as a result of alcohol use. Considering the higher rate of bai jiu use and the choice of high-ABV bai jiu in Hubei, it was noteworthy that pushing others to drink and being pushed was lowest in Hubei. This suggested to us a pattern of use consistent with Confucian values. Our findings indicated possible cultural controls for alcohol use. Two studies of Chinese young adults have found a correlation between a stronger Chinese cultural orientation and alcohol behaviors [23,28], again suggesting the importance of cultural traditions.

While unrecorded bai jiu drinking was more common in Hubei, the frequency of drinking was higher in Hebei, where unrecorded bai jiu drinkers were more likely to consume spirits every day than unrecorded bai jiu drinkers in the other two provinces. On memorable occasions, bai jiu drinkers in Hebei and Anhui consumed more bai jiu than their counterparts in Hubei. There is no obvious explanation for this difference other than Hebei borders the Beijing-Tianjin metropolitan area, which suggested the possibility that more regular traveling between rural and urban areas may have affected patterns. Hebei is also the most northern of the three provinces. Some studies have provided evidence that drinking is more common in Northern China [27].

The Hebei and Anhui sample reported a higher likelihood of experiencing social pressure in drinking. Our data showed that rural residents in Hebei and Anhui were more likely to experience being pushed or pushing others to drink. In certain social situations in China, there is significant social pressure for everyone present to drink. Under these circumstances, alcohol use is somewhat regulated by custom and etiquette of “toasting”, requiring one to drink with and toast others to show respect, affirm friendships, or indicate hospitality. Our findings suggest the need to know more about culturally specific norms in drinking in Hebei and Anhui. In addition, as suggested here, policymakers should use caution to develop policies that fit the local social and cultural context.

### Limitations

These results suggest important regional differences in patterns of unrecorded bai jiu use. As a banal part of the daily diet, alcohol use might be subject to underreporting. This may have affected the individual’s report of unrecorded and recorded bai jiu differently. Additionally, this study was performed in three provinces in rural China and only focused on bai jiu drinkers who agreed to be interviewed. Given the regional level, these results are difficultly generalizable to other settings and other countries. A more formal true experimental sampling design would produce more accurate estimates of drinking and drinking differences among the provinces. The measures used would benefit from revision, and this entire line of inquiry would benefit from a clear statement of intent as to why there could be major regional differences and why they are important. The availability of large China databases makes this a feasible undertaking (large databases include the China Kadoori Biobank Data; the China Health and Nutrition Survey Data; the China Health and Retirement Longitudinal Study Data; and the China Family Panel Study Data).

## 5. Conclusions

In a country as large and complex as China, there is a need for a better understanding of Chinese drinking patterns, including patterns of use of the widely available artisanal unrecorded bai jiu, before developing specific policies to minimize alcohol risk. This analysis found significant variations in patterns of bai jiu use (both recorded and recorded bai jiu) by province, suggesting longstanding local practices associated with the artisanal production and use of bai jiu.

Earlier work by Wei and colleagues, based on aggregated data, suggested that the use of unrecorded bai jiu was age-related (with elderly males preferring unrecorded bai jiu over recorded alcohol) and education-related, and with rural people often having less education. This study, with disaggregated samples, suggests that the role of age and education may be more nuanced.

In this analysis, age and education were not consistent in their relationship to unrecorded bai jiu use. This suggests the possibility that local cultural differences may override the effects of age and education. The present study found that rural residents of Hubei indicated a lower-risk pattern of use and begs further exploration to better understand the possible role of traditional alcohol culture. On the other hand, rural residents in Hebei and Anhui showed higher-risk drinking patterns that were possibly caused by local customs and drinking etiquette. Our findings showed that cultural traditions surrounding alcohol use may be more important in affecting drinking patterns in China than in most western countries because alcohol use is so intimately entwined in family and cultural life and because China has an alcohol use tradition that has endured for centuries [29].

## Figures and Tables

**Table 1:** Demographic characteristics of unrecorded and recorded bai jiu drinkers.

	ANHUI					HEBEI					HUBEI				
	Unrecorded		Recorded			Unrecorded		Recorded			Unrecorded		Recorded		
	*n*	%	*n*	%	χ^2^	*n*	%	*n*	%	χ^2^	*n*	%	*n*	%	χ^2^
**Sex**					3.73					2.39					1.07
Female	62	18.40	140	23.85		21	19.27	115	13.74		97	11.01	14	8.33	
Male	275	81.60	447	76.15		88	80.73	722	86.26		784	88.99	154	91.67	
**Age**					86.26 ***					70.14 ***					17.23 **
<30	16	4.75	44	7.50		4	3.67	165	19.71		32	3.63	12	7.14	
30–39	32	9.50	107	18.23		3	2.75	170	20.31		111	12.60	37	22.02	
40–49	55	16.32	204	34.75		20	18.35	192	22.93		268	30.42	50	29.76	
50–59	86	25.52	116	19.76		37	33.94	174	20.79		259	29.40	39	23.21	
≥60	148	43.92	116	19.76		45	41.28	136	16.26		211	23.95	30	17.86	
**Education**					48.63 ***					37.42 ***					19.78 **
Illiterate	89	26.41	90	15.33		18	16.51	64	7.64		29	3.29	16	9.52	
Primary or below	118	35.01	138	23.51		41	37.61	164	19.59		190	21.57	44	26.19	
Junior high	104	30.86	252	42.93		39	35.78	386	46.12		407	46.20	61	36.31	
Senior high/technical	23	6.82	99	16.87		11	10.09	177	21.15		232	26.33	46	27.38	
College or above	3	0.89	8	1.36		0 ^1^	0.00	46	5.50		23	2.61	1	0.60	
**Occupation**					47.73 ***					35.96 ***					9.39 **
Agriculture	269	79.82	316	53.83		78	71.56	390	46.59		647	73.44	131	77.98	
Service worker	25	7.42	117	19.93		8	7.34	138	16.49		120	13.62	15	8.93	
Construction	20	5.93	38	6.47		8	7.34	65	7.77		43	4.88	10	5.95	
Local worker	16	4.75	40	6.81		3	2.75	80	9.56		23	2.61	9	5.36	
Others	7	2.08	76	12.95		12	11.01	164	19.59		48	5.45	3	1.79	

Notes. ** *p* < 0.01, *** *p* < 0.001. The small cell sizes are included throughout for the purpose of describing this sample, but small cell size makes the chi-square (χ^2^) unreliable. ^1^ The chi-square for education in Hebei was not calculated because there were no participants with college or higher education.

**Table 2:** Two-level logistic regression model of the effect of province on unrecorded bai jiu drinking.

	*β*	SE	Odds Ratio	95% CI	*p*
Random Intercept	−0.18	0.66	0.83	(0.23, 3.06)	
**Province**					
Anhui	−2.90	0.68	0.06	(0.01, 0.21)	***
Hebei	−4.52	0.68	0.01	(0.00, 0.04)	***
**Male**	0.03	0.15	1.03	(0.77, 1.38)	
**Age**	0.05	0.01	1.05	(1.04, 1.06)	***
**Education**	0.02	0.07	1.02	(0.89, 1.17)	

Notes. *** *p* < 0.001. CI = confidence interval. Recorded bai jiu drinker was the reference category of the outcome in this model. Anhui and Hebei were dummy variables for province with Hubei as the reference group. Male was a dummy variable for sex, with female as the reference group. Age and education were treated as continuous variables. This model was also controlled for occupation, which was dummy coded with agriculture workers as the reference group.

**Table 3:** Two-level multinomial logistic regression model of the effect of unrecorded bai jiu drinker and province on usual drinking frequency.

	*β*	SE	Odds Ratio	95% CI	*p*
**Drinking every day** vs. **<3 times/week**					
Random Intercept	−3.91	0.41	0.02	(0.01, 0.05)	***
**Unrecorded bai jiu drinker**	1.26	0.14	3.52	(2.68, 4.61)	***
**Province**					
Anhui	0.02	0.24	1.03	(0.64, 1.63)	
Hebei	0.29	0.24	1.34	(0.83, 2.17)	
**Male**	1.92	0.16	6.83	(4.96, 9.41)	***
**Age**	0.04	0.00	1.04	(1.03, 1.05)	***
**Education**	−0.20	0.06	0.82	(0.72, 0.92)	**
**Drinking 3–6 times/week** vs. **<3 times/week**					
Random Intercept	−1.48	0.41	0.23	(0.10, 0.51)	***
**Unrecorded bai jiu drinker**	0.84	0.14	2.31	(1.76, 3.04)	***
**Province**					
Anhui	0.39	0.23	0.68	(0.43, 1.07)	
Hebei	1.13	0.25	0.32	(0.20, 0.53)	***
**Male**	1.68	0.16	5.34	(3.91, 7.30)	***
**Age**	0.00	0.00	1.00	(1.00, 1.01)	
**Education**	−0.17	0.06	0.85	(0.75, 0.96)	*

Notes. * *p* < 0.05; ** *p* < 0.01; *** *p* < 0.001. CI = confidence interval. Unrecorded bai jiu drinker was a dummy variable for drinking with recorded bai jiu drinker as the reference group. Anhui and Hebei were dummy variables for province with Hubei as the reference group. Male was a dummy variable for sex, with female as the reference group. Age and education were treated as continuous variables. This model was also controlled for occupation, which was dummy coded with agriculture workers as the reference group.

**Table 4:** Two-level linear regression model of the effect of unrecorded bai jiu drinker and province on the quantity of bai jiu consumed on daily drinking occasions.

	*β*	SE	95% CI	*p*
Random intercept	2.24	0.33	(1.60, 2.89)	***
**Unrecorded bai jiu drinker**	−0.10	0.11	(−0.31, 0.10)	
**Province**				
Anhui	−0.06	0.23	(−0.52, 0.40)	
Hebei	0.34	0.24	(−0.13, 0.81)	
**Male**	1.48	0.11	(1.26, 1.69)	***
**Age**	−0.02	0.00	(−0.02, −0.01)	***
**Education**	0.12	0.05	(0.02, 0.21)	**

Notes. ** *p* < 0.01; *** *p* < 0.001. CI = confidence interval. Unrecorded bai jiu drinker was a dummy variable for drinking with recorded bai jiu drinker as the reference group. Anhui and Hebei were dummy variables for province with Hubei as the reference group. Male was a dummy variable for sex, and female was the reference group. Age and education were treated as continuous variables. This model was also controlled for occupation, which was dummy coded with agriculture workers as the reference group.

**Table 5:** Two-level linear regression model of the effect of unrecorded bai jiu drinker and province on the quantity of bai jiu consumed on memorable occasions.

	*β*	SE	95% CI	*p*
Random Intercept	1.77	0.53	(0.74, 2.80)	***
**Unrecorded bai jiu drinker**	−0.22	0.15	(−0.52, 0.08)	
**Province**				
Anhui	0.94	0.43	(0.09, 1.79)	*
Hebei	1.99	0.44	(1.12, 2.85)	***
**Male**	2.10	0.16	(1.79, 2.42)	***
**Age**	−0.02	0.01	(−0.03, −0.01)	***
**Education**	0.21	0.07	(0.07, 0.35)	***

Notes. * *p* < 0.05; *** *p* < 0.001. CI = confidence interval. Unrecorded bai jiu drinker was a dummy variable for drinking with recorded bai jiu drinker as the reference group. Anhui and Hebei were dummy variables for province with Hubei as the reference group. Male was a dummy variable for sex, with female as the reference group. Age and education were treated as continuous variables. This model was also controlled for occupation, which was dummy coded with agriculture workers as the reference group.

**Table 6:** Two-level logistic regression model of the effect of unrecorded bai jiu drinker and province on strength of alcohol consumed on memorable occasions.

	*β*	SE	Odds Ratio	95% CI	*p*
Random Intercept	1.80	0.67	6.02	(1.60, 22.60)	**
**Unrecorded bai jiu drinker**	0.47	0.14	1.60	(1.22, 2.11)	**
**Province**					
Anhui	−1.74	0.72	0.18	(0.04, 0.72)	*
Hebei	−3.03	0.72	0.05	(0.01, 0.20)	***
**Male**	0.38	0.14	1.47	(1.11, 1.95)	**
**Age**	0.01	0.00	1.01	(1.00, 1.02)	
**Education**	−0.01	0.06	0.99	(0.88, 1.12)	

Notes. * *p* < 0.05; ** *p* < 0.01; *** *p* < 0.001. CI = confidence interval. Consuming low-strength bai jiu was the reference category of the outcome in this model. Unrecorded bai jiu drinker was a dummy variable for drinking with recorded bai jiu drinker as the reference group. Anhui and Hebei were dummy variables for province with Hubei as the reference group. Male was a dummy variable for sex, with female as the reference group. Age and education were treated as continuous variables. This model was also controlled for occupation, which was dummy coded with agriculture workers as the reference group.

**Table 7:** Two-level logistic regression model of the effect of unrecorded bai jiu drinker and province on stability of alcohol type preference.

	*β*	SE	Odds Ratio	95% CI	*p*
Random Intercept	1.06	0.37	2.89	(1.40, 5.96)	**
**Unrecorded bai jiu drinker**	−1.41	0.12	0.25	(0.19, 0.31)	***
**Province**					
Anhui	−0.62	0.26	0.54	(0.32, 0.91)	*
Hebei	−0.78	0.27	0.46	(0.27, 0.78)	**
**Male**	0.43	0.12	1.53	(1.21, 1.94)	***
**Age**	0.02	0.00	1.02	(1.01, 1.03)	***
**Education**	−0.18	0.05	0.84	(0.75, 0.93)	**

Notes. * *p* < 0.05; ** *p* < 0.01; *** *p* < 0.001. CI = confidence interval. Choosing other alcohol types was the reference category of the outcome in this model. Unrecorded bai jiu drinker was a dummy variable for drinking, and recorded bai jiu drinker was the reference group. Anhui and Hebei were dummy variables for province, and Hubei was the reference group. Male was a dummy variable for sex, and female was the reference group. Age and education were treated as continuous variables. This model was also controlled for occupation, which was dummy coded with agriculture workers as the reference group.

**Table 8:** Two-level logistic regression model of the effect of unrecorded bai jiu drinker and province on being pushed to drink.

	*β*	SE	Odds Ratio	95% CI	*p*
Random Intercept	−2.49	0.38	0.08	(0.04, 0.17)	***
**Unrecorded bai jiu drinker**	0.50	0.11	1.64	(1.31, 2.06)	***
**Province**					
Anhui	1.06	0.29	2.88	(1.62, 5.13)	***
Hebei	0.80	0.30	2.24	(1.25, 4.01)	**
**Male**	0.82	0.12	2.27	(1.80, 2.86)	***
**Age**	−0.01	0.00	0.99	(0.99, 1.00)	
**Education**	0.43	0.05	1.54	(1.39, 1.71)	***

Note. ** *p* < 0.01; *** *p* < 0.001. CI = confidence interval. Not being pushed to drink was the reference category of the outcome in this model. Unrecorded bai jiu drinker was a dummy variable for drinking with recorded bai jiu drinker as the reference group. Anhui and Hebei were dummy variables for province, with Hubei as the reference group. Male was a dummy variable for sex, with female as the reference group. Age and education were treated as continuous variables. This model was also controlled for occupation, which was dummy coded with agriculture workers as the reference group.

**Table 9:** Two-level logistic regression model of the effect of unrecorded bai jiu drinker and province on pushing others to drink.

	*β*	SE	Odds Ratio	95% CI	*p*
Random Intercept	−1.85	0.36	0.16	(0.08, 0.32)	***
**Unrecorded bai jiu drinker**	0.45	0.12	1.56	(1.25, 1.96)	***
**Province**					
Anhui	0.77	0.27	2.17	(1.28, 3.67)	**
Hebei	0.56	0.27	1.75	(1.03, 3.00)	*
**Male**	0.81	0.13	2.25	(1.75, 2.89)	***
**Age**	−0.02	0.00	0.98	(0.98, 0.99)	***
**Education**	0.25	0.05	1.28	(1.16, 1.42)	***

Note. * *p* < 0.05; ** *p* < 0.01; *** *p* < 0.001. CI = confidence interval. Not pushing others to drink was the reference category of the outcome in this model. Unrecorded bai jiu drinker was a dummy variable for drinking with recorded bai jiu drinker as the reference group. Anhui and Hebei were dummy variables for province, with Hubei as the reference group. Male was a dummy variable for sex, with female as the reference group. Age and education were treated as continuous variables. This model was also controlled for occupation, which was dummy coded with agriculture workers as the reference group.

## Data Availability

The data sets used and/or analyzed during the current study are available from the corresponding author on reasonable request.
